# High-efficiency secretory expression and characterization of the recombinant type III human-like collagen in *Pichia pastoris*

**DOI:** 10.1186/s40643-022-00605-4

**Published:** 2022-11-04

**Authors:** Zhi-Xiang Xiang, Jin-Song Gong, Jin-Hao Shi, Chun-Fang Liu, Heng Li, Chang Su, Min Jiang, Zheng-Hong Xu, Jin-Song Shi

**Affiliations:** 1 Key Laboratory of Carbohydrate Chemistry and Biotechnology, Ministry of Education, School of Life Sciences and Health Engineering, Jiangnan University, Lihu Avenue No. 1800, Wuxi, 214122 People’s Republic of China; 2 National Engineering Research Center for Cereal Fermentation and Food Biomanufacturing, School of Biotechnology, Jiangnan University, Wuxi, 214122 People’s Republic of China; 3 Jiangsu Provincial Research Center for Bioactive Product Processing Technology, Jiangnan University, Wuxi, 214122 People’s Republic of China

**Keywords:** Human-like collagen, Secretory expression, Fermentation, Protein purification, Characterization

## Abstract

Collagen, the highest content protein in the body, has irreplaceable biological functions, and it is widespread concerned in food, beauty, and medicine with great market demand. The gene encoding the recombinant type III human-like collagen α1 chain fragment was integrated into *P. pastoris* genome after partial amino acids were substituted. Combined with promoter engineering and high-density fermentation technology, soluble secretory expression with the highest yield of 1.05 g L^−1^ was achieved using two-stage feeding method, and the purity could reach 96% after affinity purification. The determination of *N*/*C*-terminal protein sequence were consistent with the theoretical expectation and showed the characteristics of Gly-X-Y repeated short peptide sequence. In amino acid analysis, glycine shared 27.02% and proline 23.92%, which were in accordance with the characteristics of collagen. Ultraviolet spectrum combined with Fourier transform infrared spectroscopy as well as mass spectrometry demonstrated that the target product conformed to the characteristics of collagen spectrums and existed as homologous dimer and trimer in the broth. This work provided a sustainable and economically viable source of the recombinant type III human-like collagen.

**Graphical Abstract:**

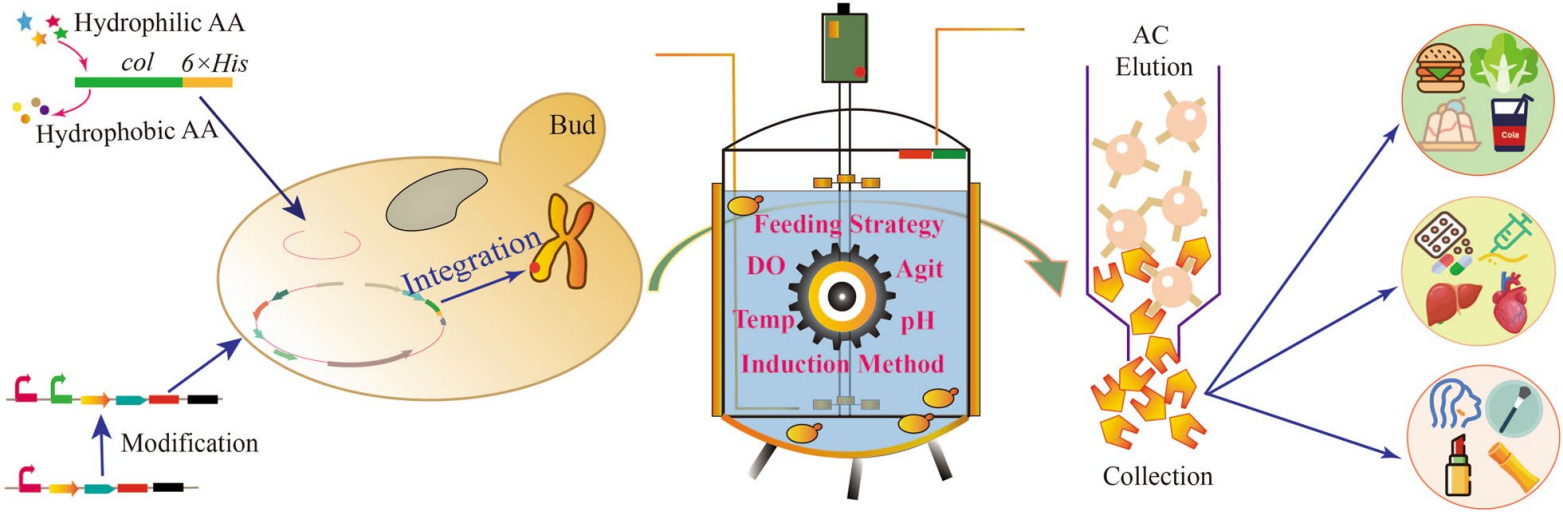

**Supplementary Information:**

The online version contains supplementary material available at 10.1186/s40643-022-00605-4.

## Introduction

Collagen is a functional structural protein widely distributed in the extracellular matrix (ECM) of the connective tissues of humans and animals, accounting for 30% of the total protein in the whole body (Muyonga et al. 2004). Three α-polypeptide chains hover around each other into the right-handed triple helix, then, assembled by 1/4 stagger and parallel arrangement to form the supermolecular aggregation structure of collagen–collagen fibril (Liu et al. 2019). The molecular composition of type III collagen is homotrimer (Wang et al. 2022), in which the (Gly-X-Y)_n_ tripeptide repeat sequence is the main feature of the spiral region.

Collagen type III, abundant in blood vessels, gut and skin, is up-regulated during the growth and wound healing of organism (Walimbe et al. 2019). In the process of wound healing, type III collagen induces the migration of inflammatory cells and fibroblasts to the wound site to promote connective tissue formation and recovery (Makuszewska et al. 2020). Type III collagen is also a natural hemostatic material, which can induce platelet adhesion and aggregation to the bleeding site and activate some coagulation factors in coagulation cascade reaction (Kuivaniemi and Tromp. 2019; Seon et al. 2017). Recently, Wang et al. (2022) asserted that recombinant human type III collagen has a practical effect in promoting ECM remodeling, upregulating the synthesis of type I and type III collagen in vivo, and alleviating skin photoaging caused by ultraviolet radiation. Collagen type III with unique physiological functions, has been widely utilized in food, beauty, and medicine, and the market demand is increasing.

Most collagen products on the market are extracted from the tissues and organs of mammals, which require a lot of raw materials, and there is a risk of pathogen contamination in applications such as prions, HIV, foot-and-mouth disease virus, etc. (Kotler et al. 2019). In addition, religious factors and ethnic differences would also be factors restricting the application (Liu et al. 2015). So, researchers turn their attention to the marine organisms (Subhan et al. 2021). However, some shortcomings, such as partial bioactivity loss, complex components, solvent residue, immunogenicity, as well as excessive content of heavy metals and toxic substances in raw materials, cannot be overcome (Xiang et al. 2021).

The biosynthesis of collagen has ushered in the dawn in the twenty-first century. It is a promising option to express collagen using animal and plant cells, yeast, and *Escherichia coli* systems, and to conduct large-scale preparation with high-density fermentation technology (Xiang et al. 2021). Tang et al. (2016) utilized *E. coli* as the host and conducted a small-scale production of human-like collagen in a 10-L bioreactor induced with 0.1 mM isopropyl-*β*-d-thiogalactopyranoside (IPTG) at 28 ℃, and finally obtained the output of 0.26 g L^−1^. IPTG was toxic to the strain, and an expression strategy using lactose with lower cost as inducer to replace it, approximately 0.7 g L^−1^ of collagen binding domain fusion proteins was finally available in a 3-L fermenter (Fruchtl et al. 2016). *E. coli* serves as the most common prokaryotic host, but its poor post-translational modification ability as well as the high possibility of forming inclusion bodies and endogenous pyrogenic have been plagued by researchers. While animal and plant cell systems are cumbersome to operate and require strict culture conditions, there is still a long way to go for industrial preparation.

As a safe and popular eukaryotic host in pharmaceutical protein expression, *P. pastoris* not only possesses complete post-translational modification function, but also avoids the formation of inclusion bodies and the production of endotoxin. As early as 2006, phospholipase C produced by *P. pastoris* was granted GRAS status by FDA (Vogl et al. 2013; Werten et al. 2019). Moreover, the biomass of *P. pastoris* can reach more than 400 g L^−1^ (wet weight) in the culture process, which indicates it is very suitable for high-density fermentation in bioreactors (Cereghino et al. 2002), and has great potential for large-scale production. In 2018, the co-expression of human collagen α1 chain and insulin gene in *P. past*oris was reported, showing that the yield was 300 mg L^−1^ and the stability of the product was improved (Mi et al. 2018). In addition, the mixed carbon fermentation strategy established by Wang et al. (2014) also exhibited good effects on collagen expression. When the GS115 strain was induced by a mixed carbon source at ration of 0.8 (glycerol/methanol), the highest collagen production can reach 1.27 g L^−1^, and the fermentation time was reduced by 50%. *P. pastoris* has been recognized as one of the most promising recombinant expression system for the commercial manufacturing of gelatin and collagen (Li et al. 2015).

However, since the natural collagen is an insoluble fibrin with large molecular weight, a large number of Gly-X-Y repeats are present in its sequence, and it is subject to strict post-modification regulation and self-assembly process during synthesis, resulting in the difficulty of collagen expression. For proteins that are hard to express, strategies such as increasing gene dosage, adaptive modification of elements, fusion expression with soluble tags and so on can promote the yield. However, molecular modifications of collagen expression have been less reported. Therefore, on the basis of maintaining the (Gly-X-Y)_n_ skeleton, we selected a segment of triple helical domain of type III human collagen α1 chain, and replaced hydrophobic amino acids other than Pro (such as Tyr, Phe, Leu, Ile, Val, Met, His, etc.) with hydrophilic residues (Asp, Asn, Glu, Gln, Lys, Thr, etc.) to improve the hydrophilicity of the target protein (gene name *col*). We integrated the *col* gene into the genome of *P. pastoris* and achieved high productivity through screening for high copy transformants, promoter engineering, and expanded cultivation. A series of characterizations were performed to explore its application potential after affinity purification. Our work provided a practicable approach for the preparation of type III human-like collagen (hlCOLIII).

## Materials and methods

### Strains and plasmids

Table 1 shows the strains and vectors used in our work. JM109 (stored in our laboratory) and GS115 (Invitrogen, California, USA) strains were used as cloning and expression hosts, respectively. GS115 is a histidine (His)-deficient strain, so the transformants can be screened in MD medium. The encoding sequence of collagen was deposited in our laboratory and employed for this study with some modification based on previous reports (Shi et al. 2022), while the pPIC9k is the expression vector stored in our laboratory.

**Table 1:** Strains and plasmids in this research

Strains/plasmids	Relevant characteristics	Applications	Sources
Strains			
*E. coli* JM109	High copy number, no modification or restriction on introduced DNA	Gene cloning host	Lab stock
*P. pastoris* GS115	Histidine-deficient and screened on MD plates	Gene expression host	Invitrogen
Plasmids			
pPIC9K	*E. coli–P. pastoris* shuttle vector carrying α-MF, G418	Gene expression vector	Lab stock
pUC57-*col*	pUC57 vector carrying target gene *col*, Amp	Gene cloning vector	Synthesized by Sangon Biotech
pPIC9K-*col*	pPIC9K carrying *col* gene and α-MF	Recombinant gene expression vector	This study
pPIC9K-P_ADH3_-*col*	pPIC9K carrying *col* gene, DH3 promoter, and α-MF	Recombinant gene expression vector	This study
pPIC9K-P_DAS1_-*col*	pPIC9K carrying *col* gene, DAS1 promoter, and α-MF	Recombinant gene expression vector	This study
pPIC9K-P_DAS2_-*col*	pPIC9K carrying *col* gene, DAS2 promoter, and α-MF	Recombinant gene expression vector	This study
pPIC9K-P_GCW14_-*col*	pPIC9K carrying *col* gene, GCW14 promoter, and α-MF	Recombinant gene expression vector	This study
pPIC9K-P_LRA3_-*col*	pPIC9K carrying *col* gene, LAR3 promoter, and α-MF	Recombinant gene expression vector	This study
pPIC9K-P_SDH_-*col*	pPIC9K carrying *col* gene, SDH promoter, and α-MF	Recombinant gene expression vector	This study
pPIC9K-P_GAP_-*col*	pPIC9K carrying *col* gene, GAP promoter, and α-MF	Recombinant gene expression vector	This study

### Recombinant vector construction and transformation

Using the pUC57-*col* as template and the IF/IR-*col* as primers (Additional file 1: Table S1), the target gene was amplified under the action of DNA polymerase (Phanta Flash Master Mix, Vazyme, Nanjing, China). After the objective gene and the pPIC9K were both digested by the *Eco*R I and *Not* I, the pPIC9k-*col* was ligated in a molar ratio of 7/1 under the catalysis of T4 DNA ligase and transformed into the JM109 for cloning.

The pPIC9k-*col* was extracted from the JM109/pPIC9k-*col* and linearized by *Sac* I. Some 2000 ng of linearized plasmid was added into GS115 competent cells and moved to electroporation cuvette, shocked at 2000 mV, 5 ms. 1 mL of precooled 1 M D-sorbitol was added immediately, recovered in a 220 rpm shaker at 30 ℃ for 2 h, and spread on MD plates (1.34% yeast nitrogen base, 2% glucose, and 2% agar).

### Screening of transformants with high gene dosage

The recombinant vector was integrated into the AOX1 region of the GS115 genome after being linearized, and the number of insertions was random, which leads to the difference of gene dosage among each transformant. Previous reports indicated that the increase of gene copy number is associated with elevated target protein production (Yang and Zhang. 2018; Zheng et al. 2014). Therefore, it is necessary to screen transformants with high gene dosage under the action of a high concentration of geneticin (G418) to improve protein yield. The transformants on the MD plate were picked and inoculated on YPD plates containing 1.0, 2.0, 3.0, 4.0, and 5.0 mg mL^−1^ G418, respectively, cultured at 30℃ and selected the strains that grew well.

High gene copy strains were selected and cultured in 10 mL YPD medium (1% yeast extract, 2% peptone, 2% glucose) at 30℃, 220 rpm for 18 h, and then inoculated in 25 mL BMGY medium (1% glycerin, 2% peptone, 1% yeast extract, 0.4% K_2_HPO_4_ 3H_2_O, 1.18% KH_2_PO_4_, 1.34% YNB) at 30℃, 220 rpm until *OD*_600_ is 5. After the cells were collected and washed by sterile water, they were inoculated into 25 mL fresh BMMY medium (0.5% methanol, 2% peptone, 1% yeast extract, 0.4% K_2_HPO_4_·3H_2_O, 1.18% KH_2_PO_4_, 1.34% YNB) and cultured at 30 ℃, 220 rpm, and 0.5% methanol was added every 12 h.

### Promoter engineering

The promoter is the control site of transcription initiation in the gene expression process. Although it does not directly encode a protein, it participates in the expression by regulating the transcription of the gene. The genome sequence of GS115 was retrieved from the NCBI database, and seven promoters including P_ADH3_ (Karaoglan et al. 2016), P_DAS1_ (Vogl and Glieder. 2013; Yurimoto et al. 2000), P_DAS2_ (Duan et al. 2018; Vogl and Glieder. 2013), P_GCW14_ (Liang et al. 2013; Yang and Zhang. 2018), P_LRA3_ (Liu et al. 2016), P_SDH_ (Periyasamy et al. 2013), and P_GAP_ (He et al. 2015; Yang et al. 2015) were selected. A homologous arm (20 bp) was added to the primer (Additional file 1: Table S1), and the GS115 genome was used as the template for PCR. The pPIC9k vector linearized by reverse PCR was ligated with the target fragment by homologous recombinase (ClonExpressII C112, Vazyme, Nanjing, China) and delivered into JM109. The transformation was carried out as described in 2.2. To ensure the same copy number integration of the target gene, transformants which grew well on G418 plate with the concentration of 1.0–5.0 mg mL^−1^ while failed to grow at 6.0 mg mL^−1^ were selected (Duan et al. 2019), and quantified the gene copy numbers by real-time PCR (RT-PCR). Transformants with the same copy numbers were selected and fermented as described in 2.3.

### Quantitative analysis of gene copy level

The genome of the recombinant strain as template was extracted by yeast genomic DNA extraction kit (Solarbio, Beijing, China). And the glyceraldehyde-3-phosphate dehydrogenase (*GAPDH*) was selected as the reference gene. The RT-PCR reactions and gene copy numbers calculation were carried out according to Hu et al. (2013). Primers (Additional file 1: Table S2) were designed by Primer-BLAST in NCBI (https://blast.ncbi.nlm.nih.gov/Blast.cgi).

### Fed-batch fermentation

The scale-up cultivation was carried out in a 5-L bioreactor (HuiSen Bioengineering, Wuxi, China). GS115/pPIC9k-P_DAS2_-*col* strain was activated and cultured in BMGY until *OD*_600_ was around 5, and then inoculated into BSM medium [2.67% H_3_PO_4_, 0.094% CaSO_4_·2H_2_O, 1.82% K_2_SO_4_, 1.49% MgSO_4_·7H_2_O, 0.413% KOH, 4% glycerol, 0.435% PTM1 (REBIO, Shanghai, China)] with 4% inoculation quantity, adjusted rotation speed of 600 rpm min^−1^ and ventilation rate of 1 vvm, and cultured at 30 ℃, pH 5. The dissolved oxygen (DO) gradually decreased to a nadir in the initial stage and then rebounded to 80%, indicating that the original glycerol in BSM had been exhausted. In the stage of glycerol feeding, the rotating speed was increased to 800 rpm min^−1^, and 50% (*w*/*w*) glycerol containing 1.2% (*v*/*v*) PTM1 was fed at a rate of 18 mL h^−1^ L^−1^ until the wet weight of biomass reached some 200 g L^−1^, and the DO rebounded again to 80% after glycerol depletion. After starving for 2 h to deplete the carbon source completely, cells were subjected to the methanol induced feeding phase at pH 6.0, which was performed by enlarging the ventilation and rotation speed to 2–3 vvm and 1000 rpm min^−1^, respectively, and feeding methanol containing 1.2% (*v*/*v*) PTM1 for induction. Within the initial 4 h of induction, the cells experienced an adaptive process to methanol, and the feeding interval is controlled to supplement 1 s every 100 s (0.22 mL s^−1^). Afterward, the feeding speed was gradually increased according to the pulse feeding strategy that feeding was performed when the DO rose back to 40%. The DO remained above 20% in the whole induction stage, and the fermentation ended after 96 h.

### SDS-PAGE and western blotting

The expression of hlCOLIII was analyzed by SDS-PAGE using 12% Tris-Gly resolving gels according to Laemmli (1970), stained with Coomassie Brilliant Blue G250. The densitometric value of the desired band in the destained treated gel were analyzed by ImageJ software, and compared to the protein ladder (BBI, Shanghai, China) with known concentration (Alonso Villela et al. 2020; Peng et al. 2014; Wang et al. 2014). The protein on the gel was migrated onto polyvinylidene fluoride (PVDF) by wet electrophoretic transfer and blocked with 5% bovine serum albumin (BSA) for 2 h. After extensive washing with TBST (150 mM NaCl, 20 mM Tris–HCl, 0.1% Tween-20), the PVDF was incubated with the primary antibody (anti-6 × His tag antibody) and secondary antibody (HRP-conjugated goat anti-mouse lgG) sequentially at room temperature. Finally, the membrane for immunoblotting was developed with ECL reagents (ThermoFisher Scientific, MA, America).

### Purification

Ni^2+^ would chelate with the imidazole ring in the His tag, and the purpose of protein elution is achieved by replacing the 6 × His tag with an analog (glyoxaline) (Wang et al. 2009). The supernatant of the fermentation broth was treated by a 0.22 μm filter membrane and then immobilized metal ion affinity chromatography (IMAC) was carried out by AKTA protein purification system with a nickel column (His Trap HP 1 mL). After a linear gradient of glyoxaline (0–500 mM) at a flow rate of 1 mL min^−1^, samples at the peak position were collected and verified by SDS-PAGE. The purified fraction was desalted in PBS (pH 7.4) by dialysis bag (MW: 1000 Da) to obtain the pure hlCOL.

### Characterization of hlCOLIII

#### Sequencing of *N*/*C*-terminal amino acids

The samples were treated with 10 mM dithiothreitol (DTT) and 50 mM iodoacetamide (IAA) for reductive alkylation, and then digested by trypsin and staphylococcus Glu-C to obtain peptide fragments, which were subjected to determination by hybrid ion trap-orbitrap mass spectrometer (Orbitrap Elite™, ThermoFisher Scientific, MA, America) combined with capillary high-performance liquid chromatography (Ultimate 3000, ThermoFisher Scientific, MA, America). Data analysis was performed using the PEAKS Studio software.

#### Amino acid analysis

The lyophilized sample of pure hlCOLIII was neutralized with NaOH after hydrolysis with 6 M HCl at 120 ℃ for 22 h. Samples were analyzed for the content of each amino acid with the automatic amino acid analyzer (S433D, sykam, Munich, Germany) after co-derivatization by o-phthalaldehyde (OPA) and fluorenyl chloroformate (FMOC-Cl). The content of each amino acid were calculated according to the area of the corresponding peak.

#### Ultraviolet spectrum scanning

200 μL of hlCOLIII aqueous solution (1 mg mL^−1^) was injected into 96-well quartz plate, and a full scan in the wavelength range of 200–400 nm was performed using a Multi-Mode Microplate Reader (SpectraMax M2e, Molecular Devices, San Francisco, America) at room temperature. SoftMax Pro 6.3 software provides data acquisition and analysis.

#### Molecular weight determination

1 μL of hlCOLIII aqueous solution and 1 μL of matrix solution (saturated *α*-cyano-4-hydroxycarboxylic acid, 50% acetonitrile, 0.1% trifluoroacetic acid, 10% acetone) were mixed and spotted onto a steel MALDI plate. After drying, the relative molecular weights were measured by matrix-assisted laser desorption ionization time-of-flight mass spectrometry (MALDI-TOF-MS, ultrafleXtreme, Bruker Daltonics, America). The mass-to-charge ratio (*m/z*) was set within the range of 5000–50000, and 200 collection peaks were superimposed per sample.

#### Analysis of Fourier transform infrared spectroscopy

The hlCOLIII is detected by Fourier transform infrared spectroscopy (FTIR) (Nexus, Thermo Nicolet, America) after being milled in a 1:100 ratio in pure KBr. The sample was put into the sample slot for tableting and scanned 32 times. The spectrum in the wavelength range of 4000–400 cm^−1^ was then collected at room temperature.

### Statistical analysis

All measurements were executed in triplicate and the data were analyzed by Microsoft Excel, expressed as mean ± standard deviation (SD). *p* < 0.05 was recognized as statistically significant.

## Results and discussion

### Construction of recombinant strains

**Fig. 1:**
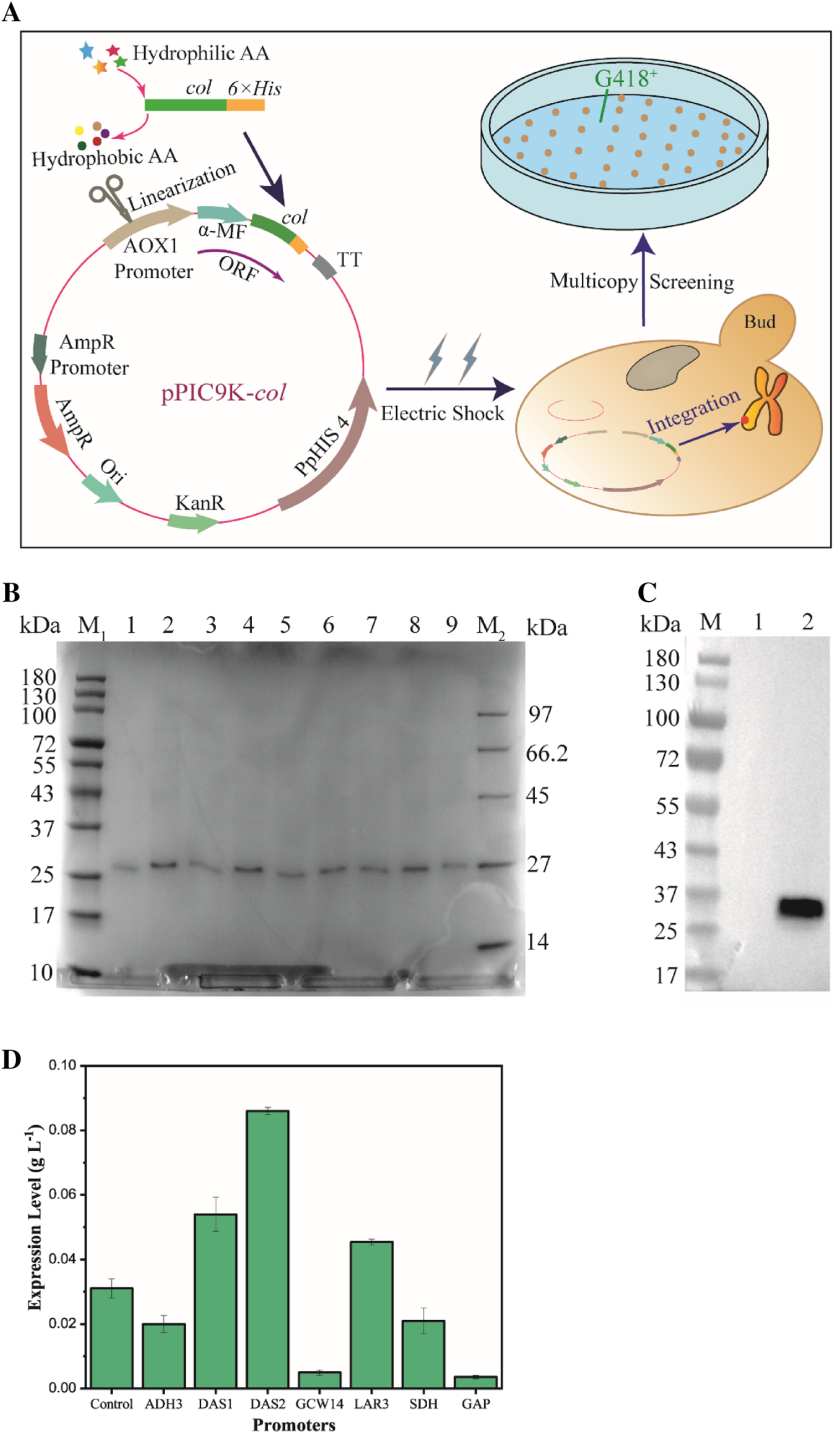
**A** The construction process of hlCOLIII high-yielding strain. The target gene after hydrophobic amino acid substitution was inserted into the pPIC9K expression vector and introduced into GS115. The transformants grown on the MD screening plate were subjected to screening for multicopy strains on plates containing high concentrations of G418. **B** SDS-PAGE analysis of 9 recombinant strains selected from the plate containing high concentration of G418 (5 mg mL^−1^). Lanes 1–9 were the expression levels of recombinant strains 1^#^–9^#^ (loading volume 20 μL, 3 times concentrated by trichloroacetic acid), respectively, as well as lanes M_1_ and M_2_ were standard molecular weights. **C** Western blotting analysis of the expression products of 2^#^ recombinant strain. Lane 1 was the blank control group (GS115/pPIC9K), lane 2 was the experimental group (2^#^ GS115/pPIC9K-*col*), and lane M was standard molecular weights. **D** The expression levels of hlCOLIII were driven by 7 different promoters in cooperation with P_AOX1_. Taking the recombinant strain GS115/pPIC9K-*col* (2^#^) as the starting strain (control)

The target gene was inserted into the multi-cloning site of the pPIC9K vector by double digestion (*Eco*R I and *Not* I), and the recombinant vector was electro-transformed into GS115 and integrated into its genome after linearization. Integrated expression can be inherited stably and save cost by eliminating the utilization of antibiotics in industrial production compared with episomal plasmid expression. The transformants were inoculated on a plate containing a high concentration of G418 to screen the multicopy clone strains. Figure 1A shows the basic process of recombinant strain construction. The genome of the target strain was extracted for PCR and sequencing verification, both of which were consistent with the expected results, indicating the recombinant strains were constructed successfully.

### Expression of hlCOLIII

Nine strains were selected from the 5.0 mg mL^−1^ G418 plate and cultured in shake flasks. SDS-PAGE gel of the fermentation broth supernatant revealed a clear band at 27 kDa (Fig. 1B, Additional file 1: Fig. S1), and strain 2^#^ displayed the highest protein expression of 0.031 g L^−1^ after 72 h of induction.

The apparent molecular weight of the target band is larger than its theoretical value (11.3 kDa), which is common in other related studies on recombinant expression of collagen (Butkowski et al. 1982; Li et al. 2015). A reasonable explanation is that there are many hydrophilic residues in collagen, which leads to the weak binding ability between protein and SDS, and the negative charge load on the target protein decreases, so the migration rate slows down and the migration distance becomes shorter (Toshihiko and Yutaka 1980). In addition, the yeast has a complete post-modification system, and the glycosylation, phosphorylation as well as hydroxylation of some residues are also the reasons for the increase of the apparent molecular weight. We performed western blotting to verify whether this band was the target protein. In Fig. 1C, there was no development in the control group, while a band appeared at 27 kDa in the experimental groups, proving that this was the target band.

### Improving the expression level with promoter modification

To increase the expression of hlCOLIII, we performed double-driving by attaching a new promoter to the back of the P_AOX1_. Among the promoters we selected, P_GCW14_ and P_GAP_ are constitutive promoters, which can be driven by only methanol when combined with P_AOX1_, whereas P_ADH3_ and P_LRA3_ need to be induced by ethanol and rhamnose, respectively, on the basis of methanol. On the basis of consistent gene copy level (Additional file 1: Fig. S2), we screened a promoter with good effect, P_DAS2_, which driven with P_AOX1_ together, and made the output of hlCOLIII reach 0.086 g L^−1^, which was 2.7 times higher than that of the original strain (Fig. 1D). We selected the strain GS115/pPIC9k-P_DAS2_-*col* for the further investigation.

### High-density fermentation

High-density fermentation is one of the most effective means to improve the output of the target product. Combined with the outstanding potential of *P. pastoris* in this regard, we carried out the lab-scale production using GS115/pPIC9k-P_DAS2_-*col* strain in a 5-L fermenter. The whole fermentation cycle was 96 h, the original glycerol in the medium was exhausted and the wet weight of the cells reached as high as 142 g L^−1^ at 24th h. The stage of glycerol supplementation began.

**Fig. 2:**
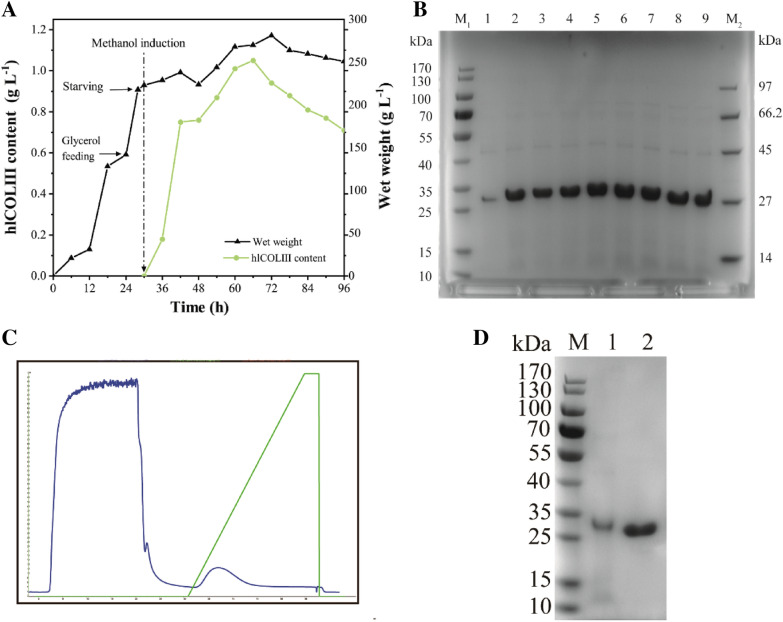
**A** High-density fermentation culture of recombinant strains was carried out in a 5-L bioreactor. Glycerol feeding for some 4 h after 24th hour of inoculation and methanol was fed at 30th h after 2 h of starvation treatment. **B** SDS-PAGE analysis of broth supernatant after 36 h of inoculation. Lanes 1–9 were cultured 36, 42, 48, 54, 60, 66, 72, 78, and 84 h (loading volume 20 μL, not concentrated), respectively, lanes M1 and M2 were protein ladder. **C** Affinity purification of expression products. **D** The purified product exhibited a single band verified by SDS-PAGE. Lane 1 was supernatant of broth before purification, lane 2 was purified product, and lane M was standard molecular weights

Glycerol is a strong inhibitor of the P_AOX1_, so the cells were starved for 2 h to completely deplete the residual glycerol as well as the acetate and other metabolites produced at the early stage. Subsequently, methanol was fed into the fermenter as both inducer and carbon source, but the content needs to be strictly controlled in the whole cycle, the toxic effect of excessive methanol can be detrimental to the whole fermentation system. According to the DO-stat, we performed pulse feeding to make methanol become the limiting factor. While the hlCOLIII was not expressed before induction, the yield reached the highest value of 1.05 g L^−1^ after at 66 h, when the cell wet weight was 270 g L^−1^ (Fig. 2A, B). After that, the biomass was still increased, but the expression of the target product began to decline, because the partial cell rupture caused the outflow of the contents, and the target protein was degraded by protease. The biomass began to decrease gradually after 72 h, and we decided to end the fermentation at 96 h.

Within 30 h after inoculation, the target product was not expressed, because P_AOX1_ and P_DAS2_ promoters both need methanol induction to initiate transcription, and the purpose at this stage is increase the biomass in preparation for subsequent induction.

Special attention should be paid to the methanol flow rate and the change of DO value during the induction phase. In the early stage of induction, it takes an adaptation process for yeast to change from glycerol feeding to methanol. At this stage, the consumption of methanol is extremely low, and the growth of biomass is slow at this stage, even the wet weight is slightly reduced due to toxic effects. Accordingly, the corresponding protein expression level was also relatively low over the 30–36th h period. However, dissolved oxygen dropped rapidly and fluctuated widely during this phase, requiring an increase in ventilation or revolution. The pulse feeding stage required adjusting the feeding rate according to the growth status and oxygen consumption of the cells. As biomass rose stepwise, methanol consumption also increased, and the expression speed reached its maximum at 36–48 h, whereas biomass decreased at 6 h thereafter, and little change in protein production might be caused by methanol overfeeding. After 66 h, due to the accumulation of ethanol, acetic acid and dead cells in the culture medium, the state of the cells was not optimal, and then the intracellular protease leaked from the broken cells, the protein accumulation gradually decreased.

### Purification

The supernatant of the broth was filtered by a 0.22 μm membrane and purified by the 6 × His tag. The purified product were collected and identified by SDS-PAGE (Fig. 2C, D). There was only one obvious electrophoretic band at 27 kDa in the whole track, the size of which was consistent with the previously validated, and there was no other miscellaneous band basically. The result of analysis suggested that the purity of the product up to about 96%, which greatly saves the process cost of downstream processing.

### Characterization

#### Sequencing of *N*/*C*-terminal amino acids

**Fig. 3:**
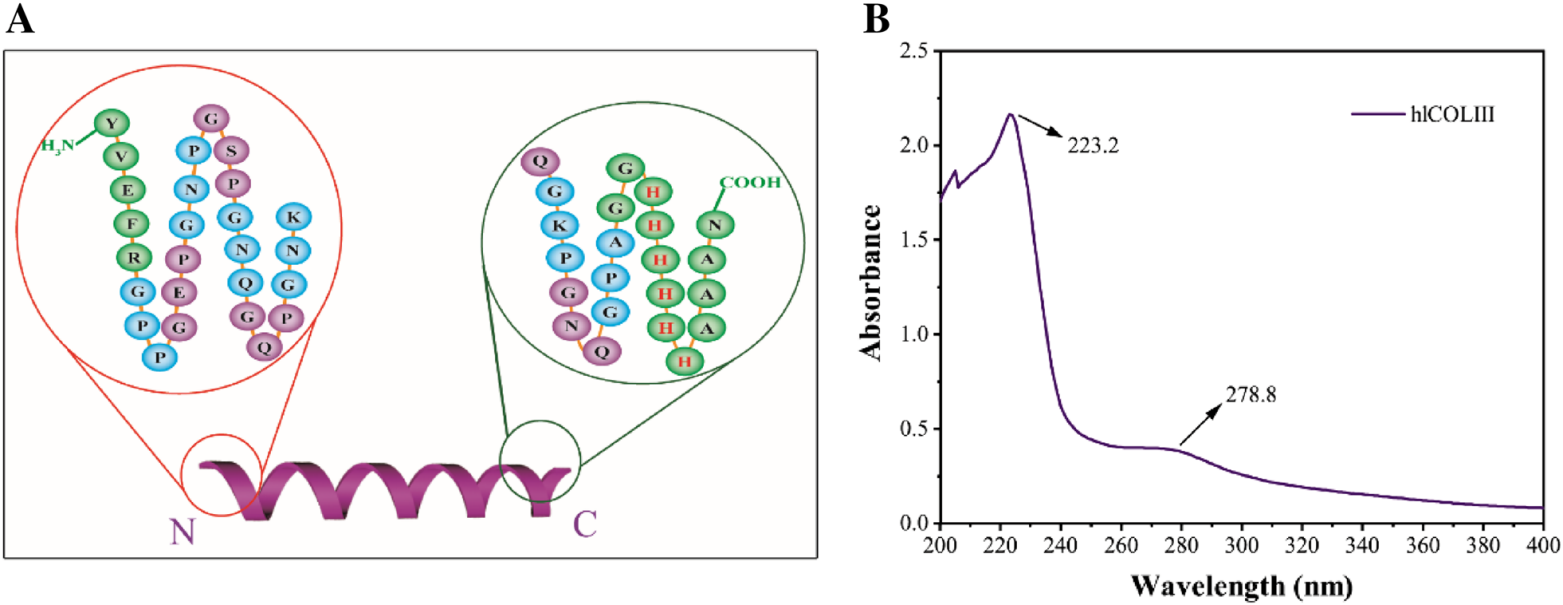
**A** The protein sequences of *N*-terminal and *C*-terminal of the expressed product. **B** The ultraviolet full-scan spectrum of hlCOLIII in the range of 200–400 nm. The absorption peaks occur at 223.2 and 278.8, respectively, due to the electronic transitions of the different groups

The results of protein sequencing at the *N*-terminal and *C*-terminal of the expressed product showed that the sequences of both were completely consistent with the theory (Fig. 3A, Additional file 1: Figs. S3, S4). The *N*-terminal sequence showed the Gly-X-Y tripeptide repeat sequences start from the 6th amino acid, and several amino acids before that were the sequence of restriction enzyme cleaving site between the signal peptide that has been excised and the target gene. Gly-X-Y also appeared in the first half of the *C*-terminal sequences, while the consecutive 6 histidine are the 6 × His tag that we added.

#### Amino acid analysis

The amino acid sequence in the triple helix region of collagen is characterized by repeating short peptides (Gly-X-Y)_n_, so, the levels of Gly and Pro should be high, which is also a distinct feature of collagen relative to other proteins. The composition and content of each residue in the expressed protein can be determined by the automatic amino acid analyzer. Table 2 lists the molar content and residue number of each component of hlCOLIII after acid hydrolysis. The Gly and Pro account for 27.02% and 23.92%, respectively, which accords with the sequence characteristics of collagen basically. The detection of Asp with Asn and Glu with Gln were reflected in their sum, so the content is high, and the existence of His was due to the 6 × His tag added in the gene design process.

**Table 2:** Amino acid analysis of hlCOLIII

Composition	Abbreviate	Assay (g/93 g)	Molar content (%)	Content (residues/1000 residues)
Aspartic acid	Asp (D)	8.26^a^	7.69^a^	77^a^
Asparagine	Asn (N)			
Glutamic acid	Glu (E)	20.82^b^	17.60^b^	176^b^
Glutamine	Gln (Q)			
Serine	Ser (S)	4.42	5.20	52
Histidine	His (H)	5.97	4.71	47
Glycine	Gly (G)	16.38	27.02	270
Threonine	Thr (T)	1.35	1.40	14
Arginine	Arg (R)	1.26	0.89	9
Alanine	Ala (A)	1.85	2.60	26
Tyrosine	Tyr (Y)	1.07	0.73	7
Cysteine	Cys (C)	0.00	0.00	0
Valine	Val (V)	1.68	1.77	18
Methionine	Met (M)	0.00	0.00	0
Phenylalanine	Phe (F)	1.48	1.11	11
Isoleucine	Ile (I)	0.00	0.00	0
Leucine	Leu (L)	0.00	0.00	0
Lysine	Lys (K)	6.30	5.33	53
Proline	Pro (P)	22.27	23.92	239

^a, b^Asn and Gln will generate Asp and Glu, respectively, after acid hydrolysis treatment, so the values in the table are Asn + Asp and Gln + Glu

#### Ultraviolet spectrum scanning

Among the 20 amino acids involved in protein synthesis, the side chain groups of aromatic amino acids (Tyr, Phe, Trp) have a photoabsorption capacity in the near ultraviolet region (200–400 nm) of electromagnetic spectrum due to the existence of benzene ring conjugated π bond system, and most proteins have absorption peak in the wavelength range of 250–280 nm (Naderi Gharagheshlagh et al. 2020). Figure 3B shows the ultraviolet spectrum of hlCOLIII in the near ultraviolet region, with two absorption peak at 223 and 278 nm, respectively, which is similar to the spectrum of natural collagen (Wu et al. 2019). The hlCOLIII contains only a small amount of Tyr and Phe (Table 2), so the absorption peak at 280 nm contributed by the transition of π → π^*^ in the benzene ring is relatively weak. The absorption peak at 210–230 nm is obviously due to the *n* → π^*^ transition of many functional groups such as –C=O, –COOH, –CONH– in the α chain of hlCOLIII (Li et al. 2020).

#### Mass spectrometry analysis

**Fig. 4:**
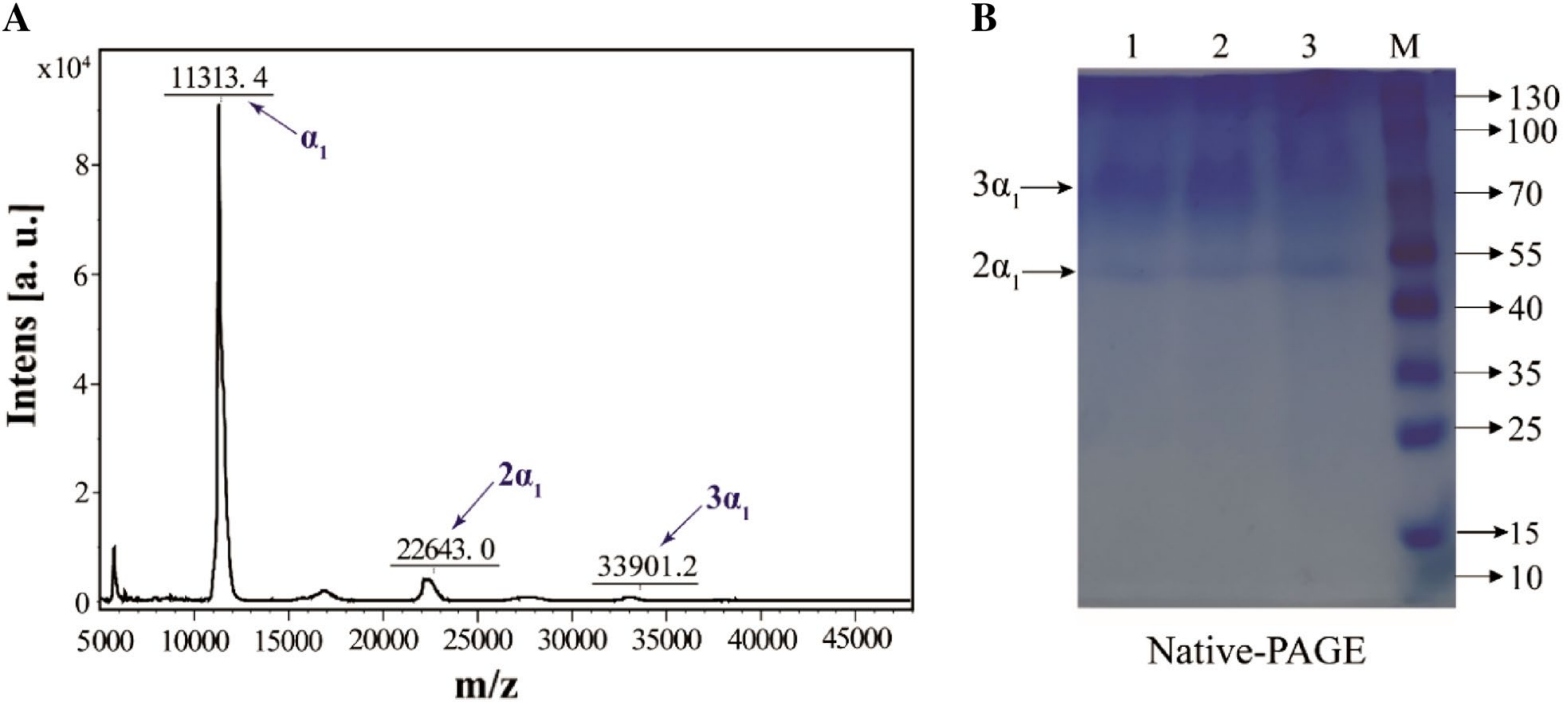
**A** MALDI-TOF-MS analysis of hlCOLIII shows that there are three peaks with decreasing intensity appeared at 11313.4, 22643.0 and 33901.2, respectively, and the *m/z* values of three were in an integral multiple relationship. **B** Native PAGE analysis of hlCOLIII. Lanes 1–3: hlCOLIII samples, with one band at 55 kDa and one between 70 and 100 kDa, respectively; Lane M: protein ladder

Mass spectrometric identification of the purified product was performed using MALDI-TOF-MS to determine the actual molecular mass. It can be seen from Fig. 4A that the molecular mass of the α1 chain of the recombinant collagen is 11.3 kDa, which is consistent with the theoretical (11.3 kDa). It is worth mentioning that there is a small peak at the mass-to-charge ratio of 22643 and 33901, whose molecular weight is twice and three times that of the α1 chain, respectively. It is speculated that the dimer and trimer were formed by two or three α1 chains, and the reason for the low content may be caused by the depolymerization of dimer and trimer in the mass spectrometric experiment. In order to verify the correctness of the hypothesis, the expression products were subjected to active PAGE electrophoresis analysis, in other words, the depolymerization effect of SDS on the protein was removed during electrophoresis, and the active expression products were directly separated in Native-PAGE. Two bands were concentrated at approximately 55 and 80 kDa (Fig. 4B, Additional file 1: Fig. S5), which were twice and three times as large as the band size in SDS-PAGE (27 kDa), respectively, indicating that hlCOLIII existed as α1 dimer and trimer in supernatant. Several other small peaks in Fig. 4A are caused by ion fragments.

#### Analysis of Fourier transform infrared spectroscopy

**Fig. 5:**
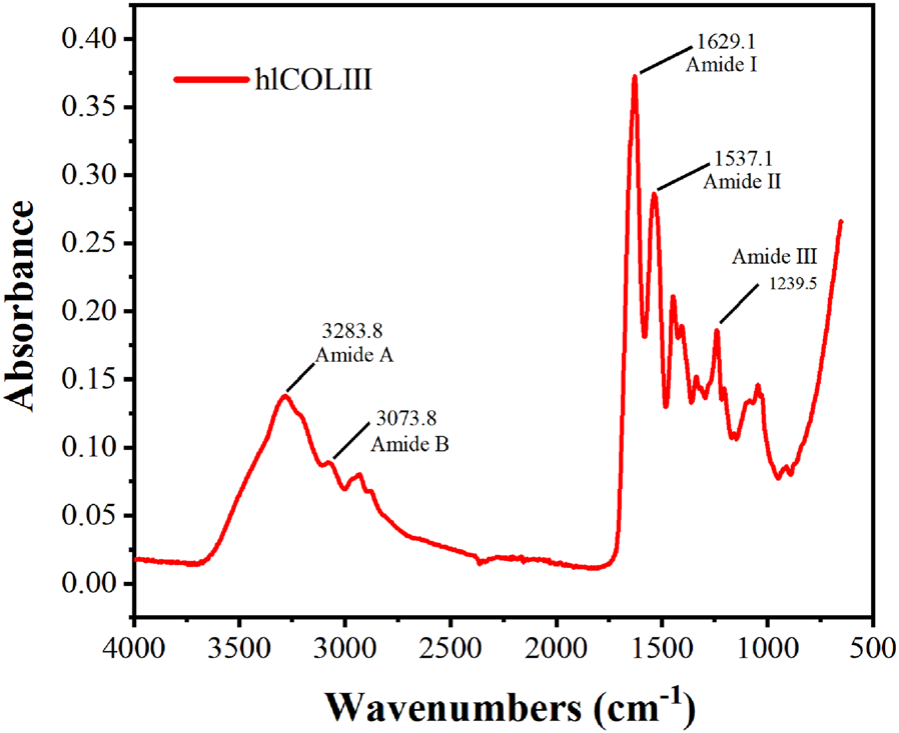
FTIR spectra of hlCOLIII displaying absorption peaks of amide A, amide B, and amide I–III

Natural collagen spirals three α chains into a triple helix structure through the action of inter and intra-chain hydrogen bonds, and its amide A, amide I, and amide III display distinct spectral characteristics. Figure 5 shows the FTIR chromatogram of hlCOLIII. The spectral characteristics and wavenumber of amide A and amide B bands in the hydrogen bond region, amide I as well as amide II in the double bond region, and amide III band located in the fingerprint region are close to those of natural collagen (Ashraf et al. 2022). Hence, hlCOLIII has similar structural features with natural collagen. The amide A band is sensitive to conformational changes in the triple helical structure of collagen, and its peak position correlates with the strength of hydrogen bonds. The amide A band generally appears in the range of 3330–3325 cm^−1^, which results from the stretching vibration of N–H bonds, and the peak position shifts to lower wavenumbers when N–H bonds participate in hydrogen bond formation. We found an amide A band around 3283.8 cm^−1^, proving that hlCOLIII has more N–H bonds involved in hydrogen bond formation than native collagen, which may affect triple helix formation.

The amide I band (1600–1700 cm^−1^) is the most clearly peptide bond vibration, which can generate a strong signal and is sensitive to protein secondary structure, but is influenced by water bending mode easily (Stani et al. 2020). The amide I band of hlCOLIII is near 1629.1 cm^−1^, which is mainly contributed by C=O bond stretching vibration, coupled C–N stretching as well as C–H bending. The amide II (1600–1500 cm^−1^), is mainly affected by N–H bending vibration as well as C–N stretching vibration in amide bonds, but less sensitive to protein conformational changes. The existence of the predominant band at 1550 cm^−1^ was attributed to natural triple helix collagen (Sizeland et al. 2018), whereas amide II was found near 1537.1 cm^−1^ in hlCOLIII. The resolution of the collagen amide III band is currently incomplete. Stani et al. (2020) reduced the amide III band of collagen to a narrower range of 1300–1175 cm^−1^ and clarified that its characteristic peaks were distributed in the three main peaks around 1280, 1240, and 1202 cm^−1^, with the peak of 1240 cm^−1^ being the most intense. The hlCOLIII has three characteristic peaks at 1279, 1239, and 1205 cm^−1^, and the absorption peak near 1239.5 cm^−1^ is the most intense, which was caused by N–H stretching vibration.

## Conclusions

To sum up, the soluble secretory expression of hlCOLIII was successfully performed by using the *P. pastoris* system in our research, which greatly simplified the downstream process and could achieve a yield of 1.05 g L^−1^. A series of characterization experiments suggested that the target protein had the structural characteristics of collagen and existed in the form of dimer and trimer in the medium supernatant. The recombinant expression of collagen in *P. pastoris* could represent a sustainable and economically viable source of the polymer, and this work laid a foundation for the industrial production of recombinant collagen.

### Supplementary Information

**Additional file 1: Table S1.** List of primers involved in this work. **Table S2.** List of primers involved in RT-PCR. **Fig. S1.** SDS-PAGE analysis of 2^#^ recombinant strain. Lanes 1-3 were the blank control (GS115/pPIC9K), lanes 4-6 were the 2^#^ recombinant strain (GS115/pPIC9K-*col*), and lane M was standard molecular weights. **Fig. S2.** Target gene copy number of double-driving recombinant strain. **Fig. S3.** *N*-terminal sequencing results of the target protein. **Fig. S4.** *C*-terminal sequencing results of the target protein. **Fig. S5.** Western blotting analysis of the native hlCOLIII (after Native-PAGE). Lane 1 was the hlCOLIII, and lane M was standard molecular weights.

## Data Availability

All data generated or analyzed during this study are included in this published article.

## Abbreviations

ECM: Extracellular matrix
IPTG: Isopropyl-*β*-d-thiogalactopyranoside
hlCOLIII: Type III human-like collagen
DO: Dissolved oxygen
G418: Geneticin
DTT: Dithiothreitol
IAA: Iodoacetamide
IMAC: Immobilized metal ion affinity chromatography
OPA: O-Phthalaldehyde
MALDI-TOF-MS: Matrix-assisted laser desorption ionization time-of-flight mass spectrometry
FMOC-Cl: Fluorenyl chloroformate
FTIR: Fourier transform infrared spectroscopy
