# Supplementary material for: High-efficiency secretory expression and characterization of the recombinant type III human-like collagen in Pichia pastoris
Source: Bioresour Bioprocess. 2022 Nov 4;9(1):117. doi: 10.1186/s40643-022-00605-4 (PMC10992891; doi:10.1186/s40643-022-00605-4)
Supplement: Supplementary file 1 — Additional file 1: Table S1. List of primers involved in this work. Table S2. List of primers involved in RT-PCR. Fig. S1. SDS-PAGE analysis of 2# recombinant strain. Lanes 1-3 were the blank control (GS115/pPIC9K), lanes 4-6 were the 2# recombinant strain (GS115/pPIC9K-col), and lane M was standard molecular weights. Fig. S2. Target gene copy number of double-driving recombinant strain. Fig. S3. N-terminal sequencing results of the target protein. Fig. S4. C-terminal sequencing results of the target protein. Fig. S5. Western blotting analysis of the native hlCOLIII (after Native-PAGE). Lane 1 was the hlCOLIII, and lane M was standard molecular weights. [file 40643_2022_605_MOESM1_ESM.docx]

**Supporting Information**

Table S1. List of primers involved in this work

| Name | Nucleotide sequence (5’→3’) |
| --- | --- |
| IF-*col* | CGGAATTCAGAGGTCCACCCGGTGAGC (*Eco*R I) |
| IR-*col* | TTGCGGCCGCATGGTGATGGTGATGATGACCACCGGCTGGACCTTG (*Not* I) |
| IF-PADH3 | aactaattattcgaaggatccCGCACCCCCAATGATCACA |
| IR-PADH3 | gaaatctcatcgtttggatccTTTCGTAAAGTAAATAAGATAAAAGCTAGTAGC |
| IF-PDAS1 | aactaattattcgaaggatccAGCAATGATATAAACAACAATTGAGTGA |
| IR-PDAS1 | gaaatctcatcgtttggatccTTTGTTCGATTATTCTCCAGATAAAAT |
| IF-PDAS2 | aactaattattcgaaggatccAATGATATTTGAGGGTGTTAGTTACTTCG |
| IR-PDAS2 | gaaatctcatcgtttggatccTTTTGATGTTTGATAGTTTGATAAGAGTG |
| IF-PGCW14 | aactaattattcgaaggatccCAGGTGAACCCACCTAACTATTTTT |
| IR-PGCW14 | gaaatctcatcgtttggatccTTTTGTTGTTGAGTGAAGCGAGTG |
| IF-PLRA3 | aactaattattcgaaggatccAATTTCGGAAAAACTTTTGGAATT |
| IR-PLRA3 | gaaatctcatcgtttggatccATTTTTAGGAGATAAAAATTCTGGGG |
| IF-PSDH | aactaattattcgaaggatccAAGTTGTATATTATTAATGGCGGGG |
| IR-PSDH | gaaatctcatcgtttggatccGTTGGATAATAGTGAGTGTAATGAAGCG |
| IF-PGAP | aactaattattcgaaggatccAGATCTTTTTTGTAGAAATGTCTTGGTG |
| IR-PGAP | gaaatctcatcgtttggatccATAGTTGTTCAATTGATTGAAATAGGG |
| Note: the lowercase parts are sequence of homologous arm (20 bp). | |

Table S2. List of primers involved in RT-PCR

| Name | Nucleotide sequence (5’→3’) |
| --- | --- |
| IFRT-*col* | AGCCTGGATCTAACGGTCCT |
| IRRT-*col* | GGCTTACCTTGCTCTCCAGG |
| IFRT-*GAPDH* | TTGACGGTCCATCCCACAAG |
| IRRT-*GAPDH* | CCTTAGCAGCACCAGTGGAA |


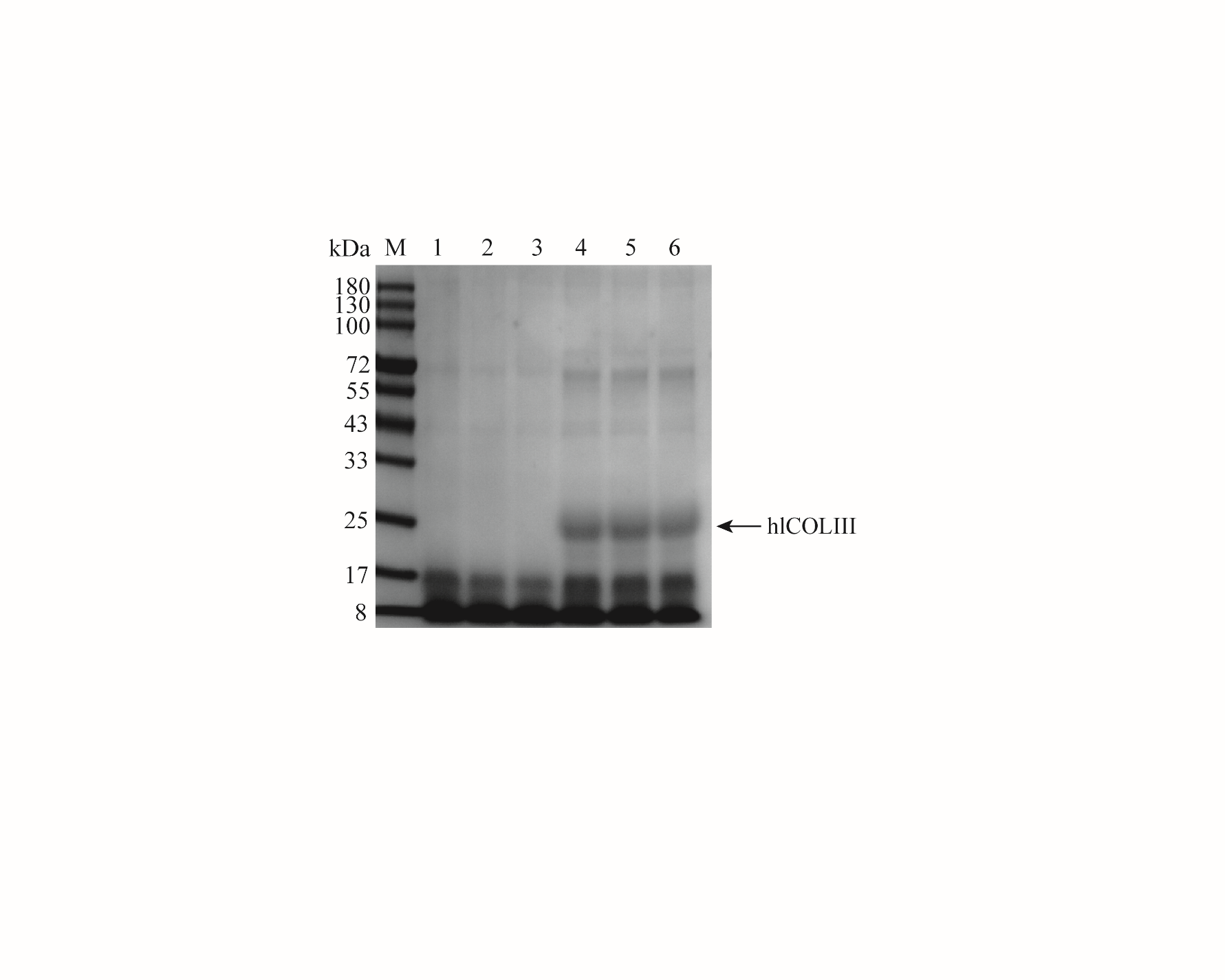


**Fig. S1.** SDS-PAGE analysis of 2^#^ recombinant strain. Lanes 1-3 were the blank control (GS115/pPIC9K), lanes 4-6 were the 2^#^ recombinant strain (GS115/pPIC9K-*col*), and lane M was standard molecular weights.


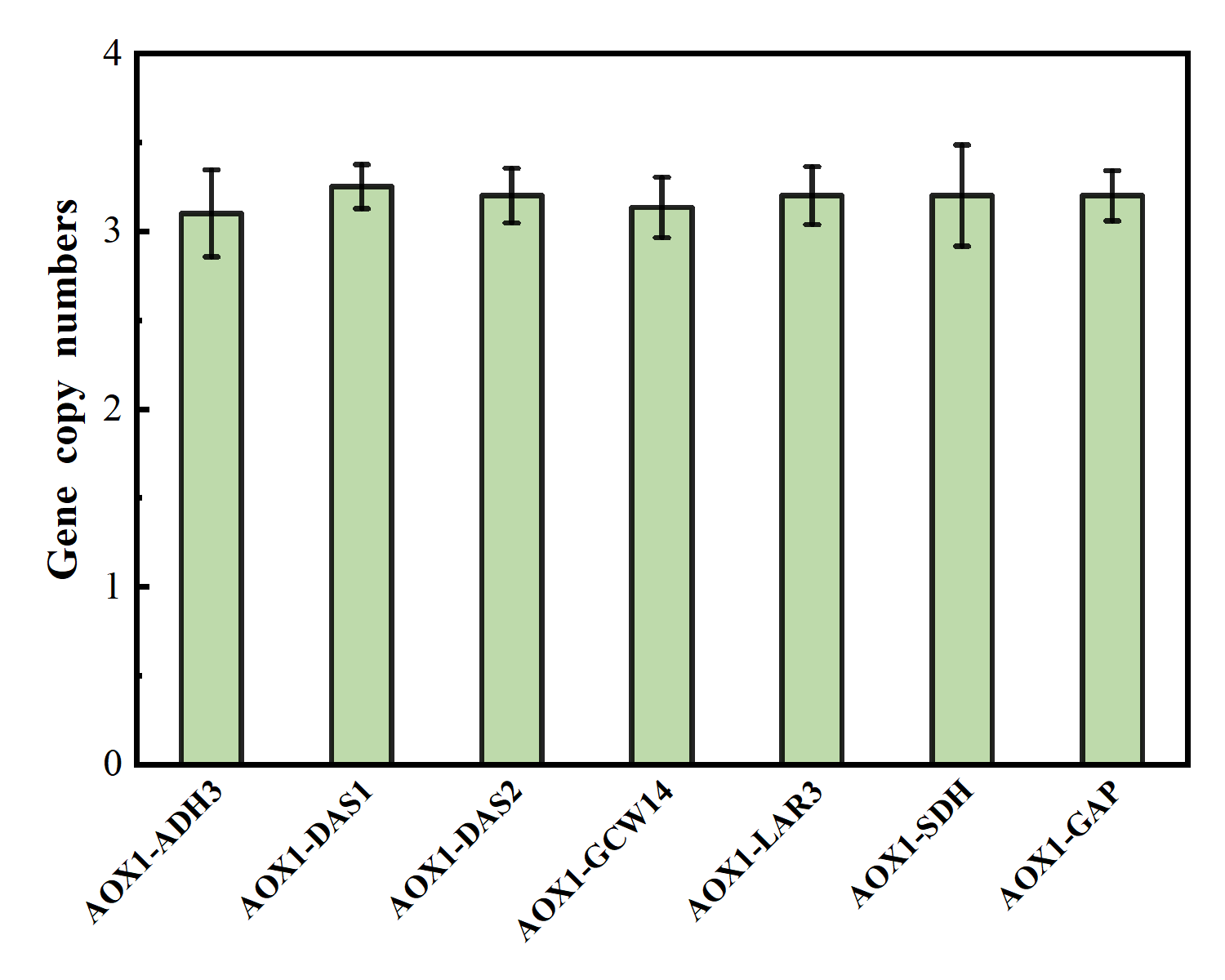


**Fig. S2.** Target gene copy number of double-driving recombinant strain.

**
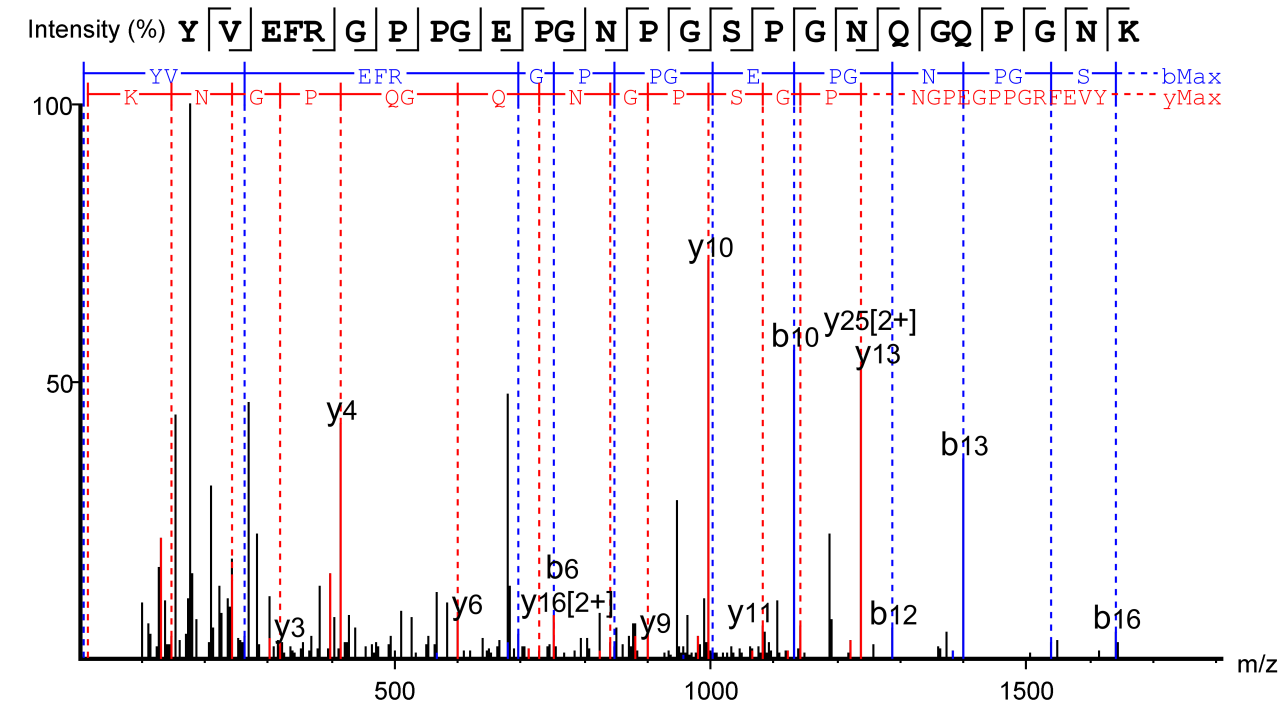
Fig.** **S3.** *N*-terminal sequencing results of the target protein.

*H_3_N*-Y-V-E-F-R-G-P-P-G-E-P-G-N-P-G-S-P-G-N-Q-G-Q-P-G-N-K…


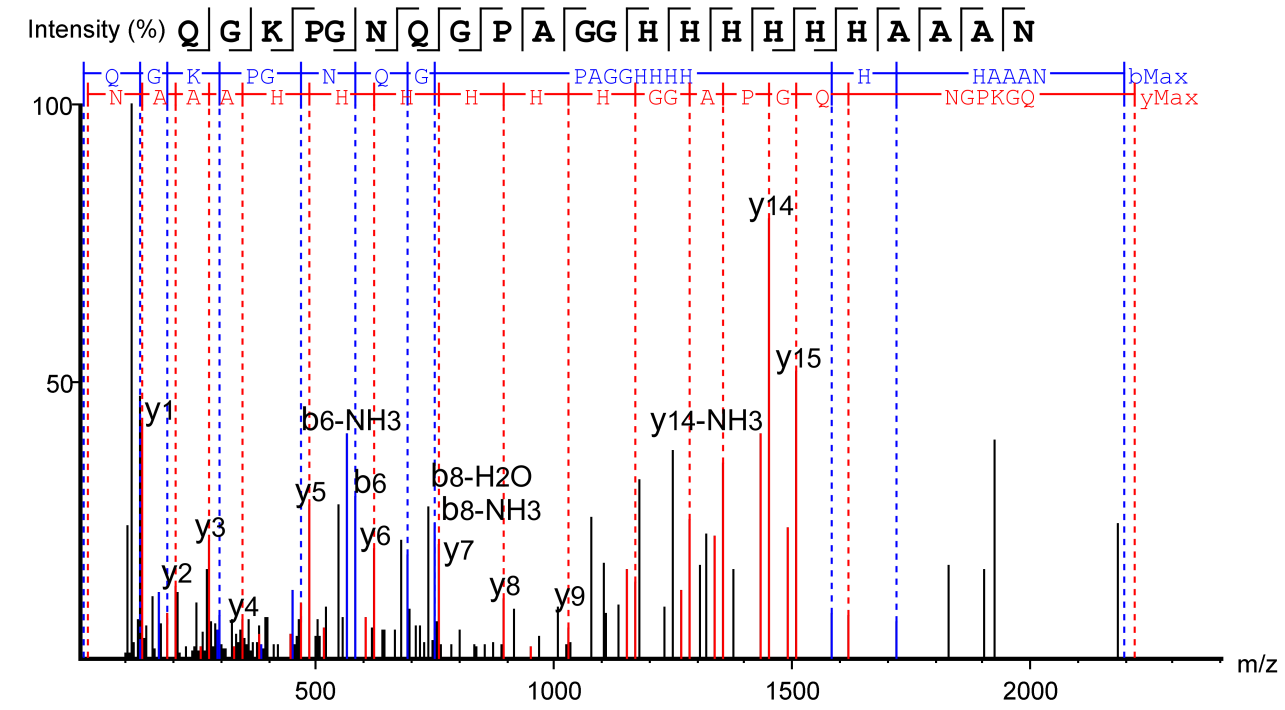
**Fig.** **S4.** *C*-terminal sequencing results of the target protein

…Q-G-K-P-G-N-Q-G-P-A-G-G-H-H-H-H-H-H-A-A-A-N-*COOH*


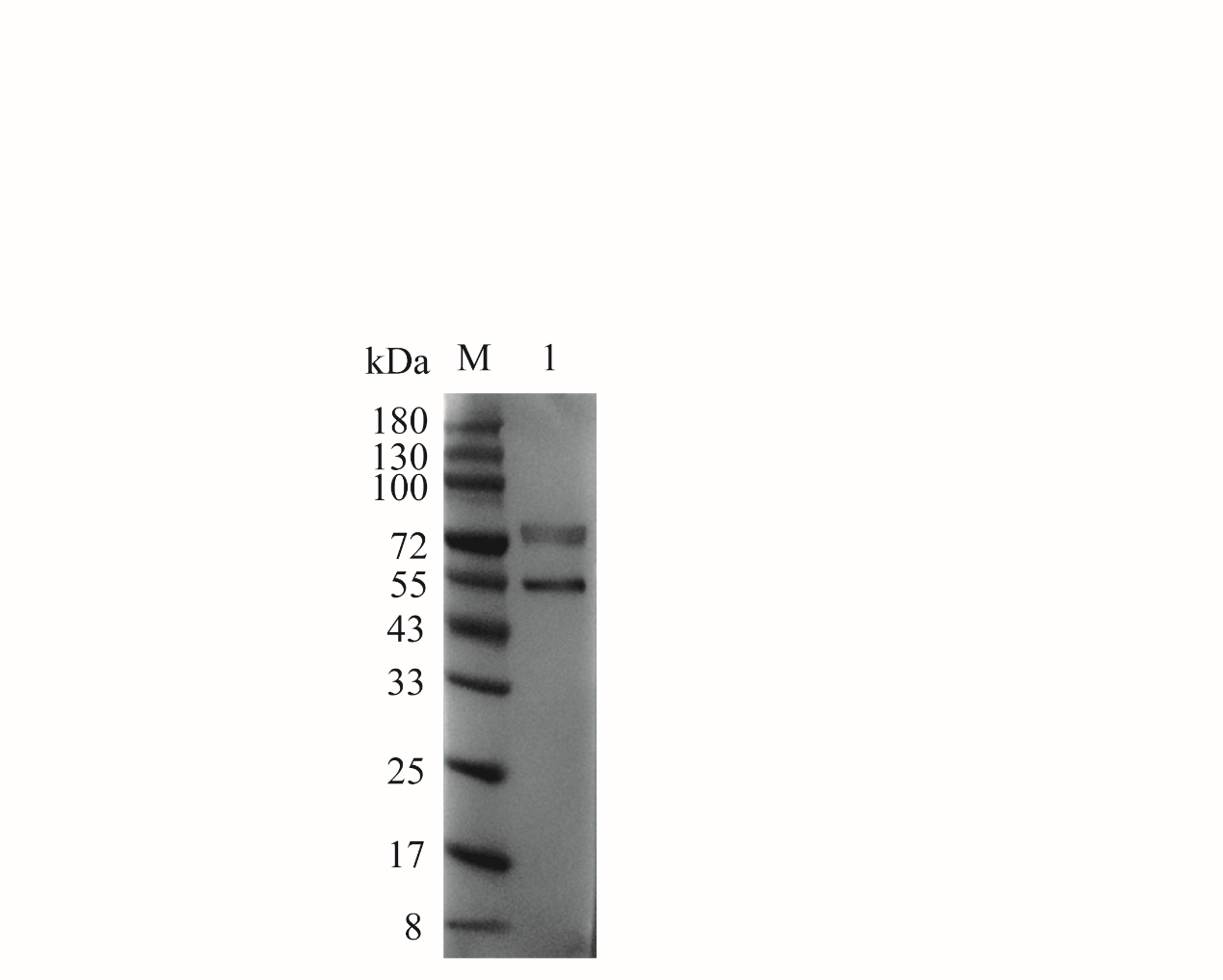


**Fig. S5.** Western blotting analysis of the native hlCOLⅢ (after Native-PAGE). Lane 1 was the hlCOLⅢ, and lane M was standard molecular weights.
